# Genome-Wide Association Analysis for Stem Cross Section Properties, Height and Heading Date in a Collection of Spanish Durum Wheat Landraces

**DOI:** 10.3390/plants10061123

**Published:** 2021-06-01

**Authors:** Carmen M. Ávila, María Dolores Requena-Ramírez, Cristina Rodríguez-Suárez, Fernando Flores, Josefina C. Sillero, Sergio G. Atienza

**Affiliations:** 1 Área Genómica y Biotecnología, Instituto Andaluz de Investigación y Formación Agraria, Pesquera, Alimentaria y de la Producción Ecológia-Centro Alameda del Obispo, Apdo. 3092, 14080 Córdoba, Spain; josefinac.sillero@juntadeandalucia.es; 2 Instituto de Agricultura Sostenible (CSIC), Alameda del Obispo, S/N, 14004 Córdoba, Spain; mdrequena@ias.csic.es (M.D.R.-R.); crodriguez@ias.csic.es (C.R.-S.); sgatienza@ias.csic.es (S.G.A.); 3 Departamento de Ciencias Agroforestales, E.T.S.I. Campus El Carmen, Universidad de Huelva, Avda. Fuerzas Armadas, S/N, 21007 Huelva, Spain; fflores@dcaf.uhu.es

**Keywords:** durum wheat, stem, DArTSeq, WSS, lodging

## Abstract

Durum wheat landraces have a high potential for breeding but they remain underexploited due to several factors, including the insufficient evaluation of these plant materials and the lack of efficient selection tools for transferring target traits into elite backgrounds. In this work, we characterized 150 accessions of the Spanish durum wheat collection for stem cross section, height and heading date. Continuous variation and high heritabilities were recorded for the stem area, pith area, pith diameter, culm wall thickness, height and heading date. The accessions were genotyped with DArTSeq markers, which were aligned to the durum wheat ‘Svevo’ genome. The markers corresponding to genes, with a minor allele frequency above 5% and less than 10% of missing data, were used for genome-wide association scan analysis. Twenty-nine marker-trait associations (MTAs) were identified and compared with the positions of previously known QTLs. MTAs for height and heading date co-localized with the QTLs for these traits. In addition, all the MTAs for stem traits in chromosome 2B were located in the corresponding synteny regions of the markers associated with lodging in bread wheat. Finally, several MTAs for stem traits co-located with the QTL for wheat stem sawfly (WSS) resistance. The results presented herein reveal the same genomic regions in chromosome 2B are involved in the genetic control of stem traits and lodging tolerance in both durum and bread wheat. In addition, these results suggest the importance of stem traits for WSS resistance and the potential of these landraces as donors for lodging tolerance and WSS resistance enhancement. In this context, the MTAs for stem-related traits identified in this work can serve as a reference for further development of markers for the introgression of target traits into elite material.

## 1. Introduction

Plant breeding has been very successful at increasing the frequency of beneficial alleles for yield at many loci [1]. As a result, modern breeding keeps making crosses between closely related high-yielding varieties, but many beneficial alleles have undoubtedly been left behind due to the bottlenecks of domestication coupled with modern breeding [1]. This is a serious problem considering the current scenario of climate change. One concern is that the modification of the conditions that are favorable for pest/disease infestation, and/or their dispersion to areas where they were previously unknown, could result in high-yield losses or even crop devastation. In addition to this, the increasing risk of heat stress is expected to result in substantial yield losses. Indeed, both chronic increases in temperature and transient hot days are already producing large decreases in yield in Australia (reviewed by Passioura et al. [2]).

Durum wheat (*Triticum turgidum* L. spp. *durum* (Desf.) Husn.) is an important food crop with an annual production of over 40 million tons (reviewed by Sall et al. [3]). The plant genetic resources available for durum wheat breeding can be divided into four different groups: wild relatives (wild emmer wheat, *T. turgidum* spp. *dicoccoides* (Körn. Ex Asch. & Graebn.)), primitive wheats (*T. turgidum* ssp. *dicoccum* (Schrank ex Schübl.) Thell.), durum wheat landraces and modern durum wheat varieties [4]. Wild species have been used as donors of traits of interest, such as disease resistance [5], grain protein content [6] or even to develop new crops [7], but landraces are more accessible for durum wheat breeding than are primitive or wild wheats.

The potential of landraces conserved in germplasm banks is widely acknowledged, but the utilization of plant genetic resources remains under-exploited due to several factors, including the insufficient phenotyping of these landraces and the lack of efficient selection tools to overcome the potential linkage drag with undesired traits. Global efforts are underway to explore the current diversity available in durum wheat collections, including landraces [8], and to promote their utilization in plant breeding, such as the recent development of a global durum wheat panel composed of modern germplasm and landraces [4]. Local efforts remain important since landraces are well adapted to the area where they were originally selected. For instance, Spanish barley landraces outperform modern cultivars at low-production sites [9]. Furthermore, the identification of beneficial alleles using DArTSeq (Diversity Arrays Technology Sequence) genotyping, along with QTL mapping, makes an efficient marker-assisted selection for high-yield under non-optimal environmental conditions possible [10] and demonstrates the potential of local landraces for breeding.

The Spanish durum wheat genetic resources are conserved at the Centre for Plant Genetic Resources (CRF-INIA). This collection has great genetic diversity for morphological, agronomic and quality traits [11,12,13,14]. Similarly, landraces from the Iberian Peninsula (Spain and Portugal) show greater diversity in relation to wheat stem sawfly (WSS, *Cephus* spp. and *Trachelus* spp.) resistance than accessions from other origins [15]. Stem properties are responsible for WSS resistance. For instance, the proportion of the stem cutting by WSS shows a significant positive correlation with the stem diameter and the plant height and a negative correlation with the number of tillers (reviewed by Chen et al. [16]). The WSS larva produces two distinct damages. In the first place, it causes decreases in grain weight because of reduced water and nutrient translocation, as it feeds within the stem [16]. In addition, it causes yield losses due to the weakening of the stem at its base, resulting in lodging or the loss of the entire head due to stem cutting [16]. Resistant varieties contain solid stems (i.e., they have stems filled with pith). A single QTL on chromosome 3B is responsible for most of the variation in the stem solidness of common [17] and durum wheat [18]. Further diversity is available since solid stem genotypes have been recovered from crosses between hollow parents [19]. Similarly, the identification of a new QTL for WSS resistance [20] suggests that other stem traits are contributing to this resistance.

Stem traits are also related to lodging (defined as the permanent displacement of stems from their vertical growth habit) which may reduce wheat yields significantly, particularly under high fertilizer conditions. Yield reductions due to early lodging are more severe than when they happen during late grain filling. Breeding efforts have diminished the losses due to early lodging [21]. In this context, the increase in the mechanical strength of the stems is considered a potential strategy to further reduce lodging caused by mechanical failure [22]. Stem traits, such as wall thickness, solidness and stem diameter, are also associated with reduced lodging [23].

In this work, we assessed a durum wheat collection for stem cross section properties, height and heading date, and we investigated the existence of marker-trait associations (MTAs) for these traits with DArTSeq markers. Additionally, we compared the location of these markers with the position of previously described QTLs for lodging and WSS resistance to evaluate the hypothesis of the co-localization of these QTLs with stem properties.

## 2. Results and Discussion

### 2.1. Genotyping

The diversity panel of durum wheat landraces was genotyped with the DArTSeq platform (Diversity Array Technology Pty Ltd., DArT P/L, Canberra, Australia). An initial set of 115,791 presence/absence variation (PAV) markers and 77,471 single nucleotide polymorphisms (SNPs) was generated. The DArTSeq markers were aligned to the durum wheat ‘Svevo’ genome [24] using BLASTn as implemented in BLAST+. A total of 35,798 markers produced a significant match with ‘Svevo’ genes from the HC (high confidence) and LC (low confidence) models (downloaded from [25]) (Supplementary Table S1). This means that only 18.5% of markers showed significant homology to ‘Svevo’. However, this does not imply that the rest of the markers do not correspond to genes. Pan-genome studies of common wheat estimate that the dispensable genome, composed of genes that are only found in a subset or are unique to individuals, is around 42.30% of the total number of genes in wheat (reviewed by Tao et al. [26]). Genes exclusive to some lines are enriched for adaptation to the environment, while those present in all lines are enriched for essential functions [27]. Thus, future studies should consider the complete DArTSeq dataset once more reference genomes for durum wheat are available, since many of the markers without a significant match to the ‘Svevo’ genome may represent genes from the dispensable genome.

### 2.2. Phenotypic Assessment, Marker-Trait Associations and Linkage Disequilibrium

**Figure 1:**
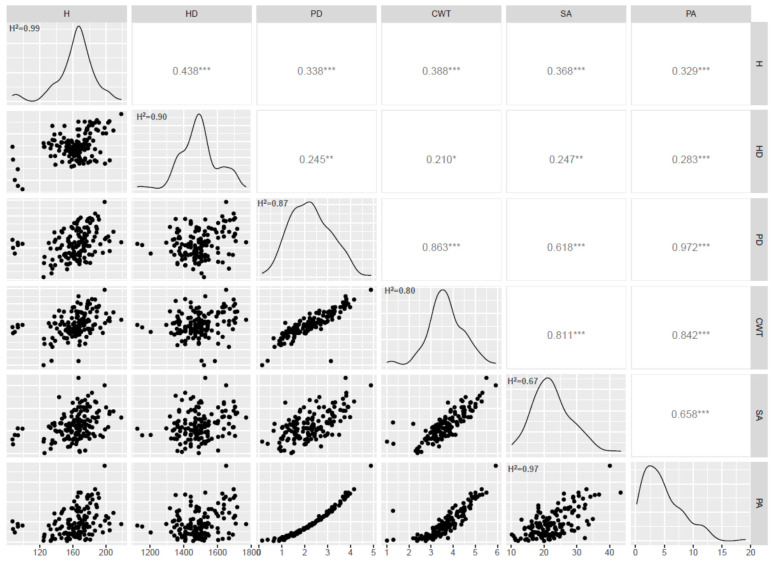
Pearson correlations and histograms showing the distribution and heritability (H^2^) of traits (BLUEs values): H = height (cm); HD = heading date (gdd); PD = pith diameter (mm); CWT = culm wall thickness (mm); SA = stem area (mm^2^); PA = pith area (mm^2^). * *p* < 0.05, ** *p* < 0.01, *** *p* < 0.001 respectively.

The screening of the durum wheat collection revealed a continuous variation for the stem area (SA), pith area (PA), pith diameter (PD), culm wall thickness (CWT), height and heading date (Figure 1). In general, all traits showed high heritability (H^2^) (Figure 1): the pith area, height and heading date showed values above 0.9; the pith diameter and the culm wall thickness had H^2^ above 0.8. The lowest H^2^ was found for the stem area (0.67). Pearson correlations were also calculated (Figure 1). The highest correlation (0.972) was obtained between the pith area and the pith diameter, as expected, since they are directly related. Correlations above 0.8 were also found between the culm wall thickness and the other stem traits.

**Figure 2:**
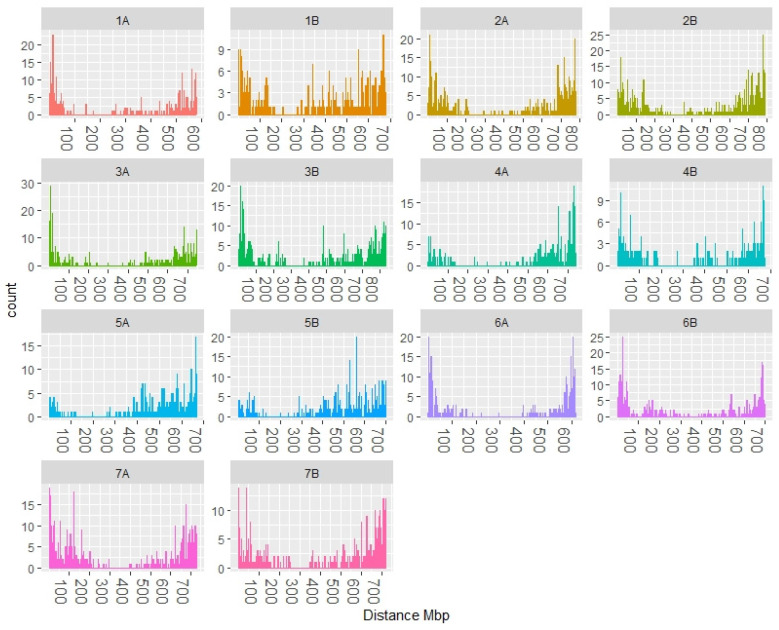
Marker distribution along the durum wheat ‘Svevo’ chromosomes.

After filtering the data to remove markers with minor allele frequencies below 5% and more than 10% of missing data, 8025 DArTSeq markers were selected for association analyses. The distribution of these markers along the wheat chromosomes was inspected by plotting their distribution at each chromosome (Figure 2).

A higher proportion of markers was detected toward the ends of chromosomes than around the centromeric regions, which is in agreement with the findings in common and durum wheat with DArTSeq markers [13]. This is important for genetic studies since the distal regions of the chromosomes show a high recombination rate and they contain the majority of the QTLs reported in durum wheat [24].

**Figure 3:**
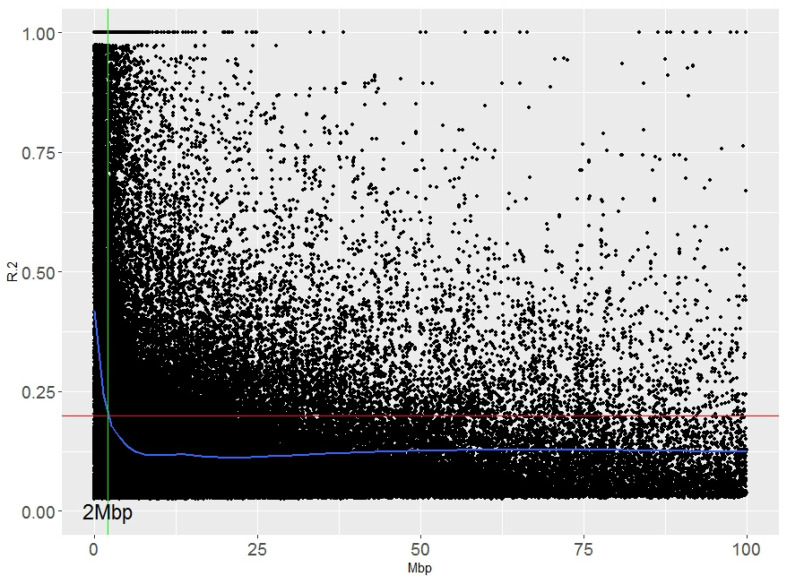
Genome-wide linkage disequilibrium (LD) decay with respect to physical distance.

Linkage disequilibrium (LD) analyses were performed that considered the whole collection of durum wheat accessions. Overall, 95% of the unlinked markers showed an r^2^ value of <0.2. This value corresponded to a distance of 2.0 Mbp (Figure 3). The LD in landraces is usually lower than in modern accessions, as revealed in the global durum wheat panel [4], with distances of 4.2 Mbp and 42.3 Mbp corresponding to the r^2^ values of the unlinked markers [4].

**Figure 4:**
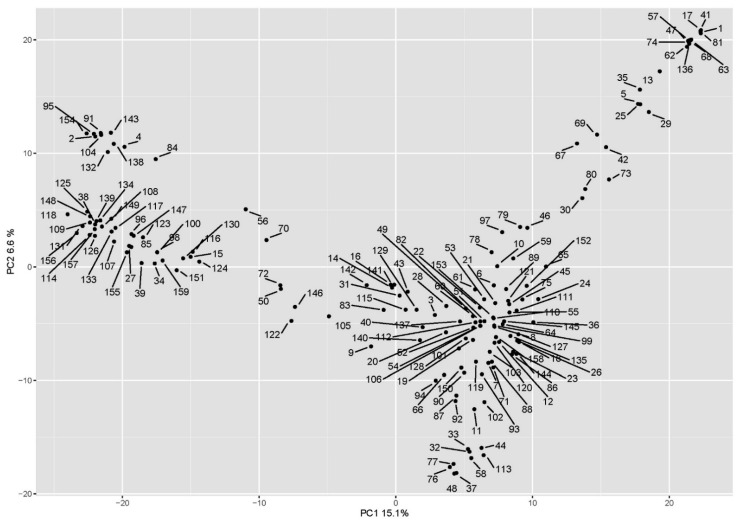
Population structure as shown from principal component analysis. Accession identifiers are shown in Table S2.

**Figure 5:**
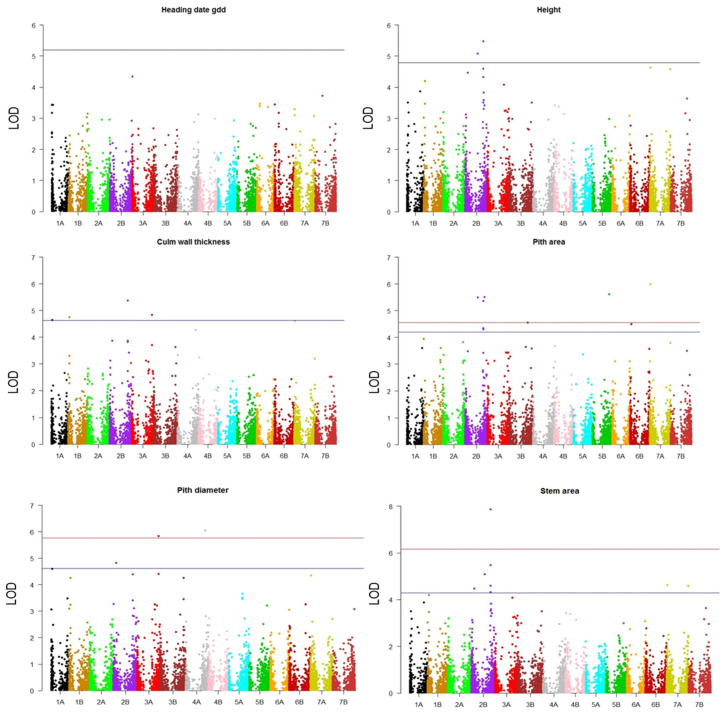
Manhattan plots from the GWAS analyses. For each trait, false discovery rate (FDR) thresholds by Benjamini and Hochberg [28] at α = 0.05 (blue horizontal line) and α = 0.01 (red horizontal line) were used to declare significant marker-trait associations (MTAs). For the heading date, the Bonferroni threshold (α = 0.05) is shown, since no significant MTAs were detected.

Phenotypic datasets were analyzed together with the marker data by genome-wide association analysis using an MLM (Q + K) model, considering the population structure matrix (the Q matrix obtained from the principal component analysis) and the kinship matrix. The biplot of the first two PC scores of the principal component analysis is shown in Figure 4. The Manhattan plots are shown in Figure 5. The marker-trait associations (MTAs) with LOD values greater than the FDR threshold of each trait were declared significant (Table 1).

A total of 29 MTAs were above the FDR threshold established for each trait and were distributed as follows: there were 2 for height on chromosomes 4A and 5A; 4 for the culm wall thickness (1A, 1B, 2B and 3A); 8 for the stem area (six on 2B and two on 7A); 4 for the pith diameter (1A, 2B, 3A and 4B) and 10 for the pith area (six on 2B, 3B, 5B, 6B and 7A). No MTA above the threshold was identified for the heading date, but the marker with the highest LOD—3570185—was selected for further inspection.

**Figure 6:**
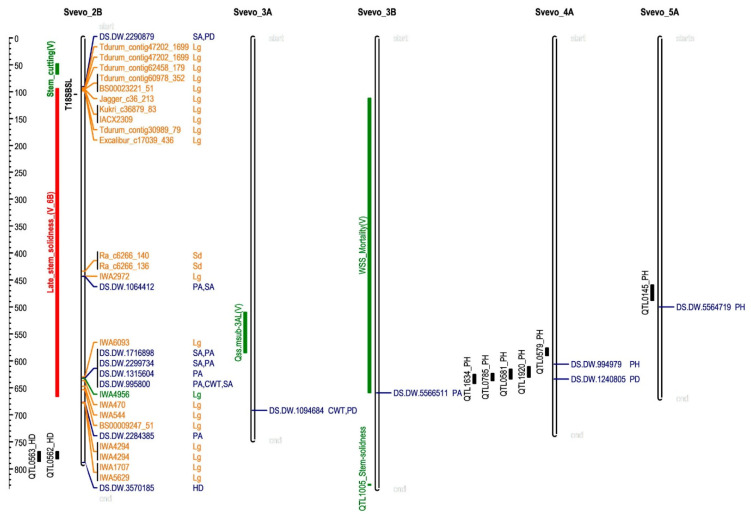
Co-localization of marker-trait associations identified in this work with previous QTLs. DS.DW., DArTSeq-durum wheat. Trait abbreviations: CWT, culm wall thickness; HD, heading date; PH, height; PA, pith area; PD, pith diameter; SA, stem area. Previously reported QTLs are shown to the left of chromosomes: in black, stem-breaking strength of the third internode at 18 days after flowering (T18SBSL) [34], heading date and plant height; in green, wheat stem sawfly, (V means QTLs reported by [20]); in red, the putative position of late stem solidness QTL according to the position of the markers within its confidence interval in chromosome 2B of the ‘Svevo’ genome (Supplementary Table S3). In orange, the position of markers associated with lodging (Lg) and stem diameter (Sd) in bread wheat (Table S4).

To explore the possibility that the MTAs identified in this work correspond to genome regions previously described, the QTL tracks available at the ‘Svevo’ genome browser [24] were downloaded and compared with the positions of the MTAs described herein (Figure 6). For height, the MTAs identified seem to co-localize with previously described QTLs (Figure 6). Indeed, the marker DS.DW.994979 is in the same region as the QTL0579 and the QTL0581 [29], the QTL0785 [30], the QTL1634 [31] and the QTL1920 [32]. The marker DS.DW.994979 is located between the QTL1920 (4 Mbp apart) and the QTL0579 (30 Mbp).

Similarly, the QTL0145 for plant height [33] is in the proximity of DS.DW.5564719 on chromosome 5A. The present work includes the Spanish durum wheat core collection, as does the work by Giraldo et al. [33], and thus, both QTLs identify the same genetic variation. Regarding the heading date, the marker scored a LOD below the FDR threshold. However, it co-localizes with two QTLs for the heading date—the QTL0562 and the QTL0563—reported by Maccaferri et al. [29], which supports the relevance of this MTA. The co-localization of the MTA identified in this work for the height and the heading date with the QTLs already reported is reassuring since these traits have been extensively studied.

Regarding stem properties, no QTL for stem traits was located in the vicinity of the areas identified in this work with the ‘Svevo’ QTL track. Thus, the GWAS track available at the bread wheat ‘Chinese Spring’ genome browser [35] was explored in looking for markers associated with lodging or stem diameter in the chromosomes where our MTAs are located. After this, the positions of these markers in the ‘Svevo’ genome were identified and compared to our results (Figure 6).

All the MTAs for stem traits in chromosome 2B co-located with markers related to lodging or stem diameter in bread wheat (Figure 6). The marker DS.DW.2290879 is related to stem area and pith diameter, and it is located at 90.2 Mbp, in close proximity to 10 markers that are related to lodging tolerance in bread wheat (located between 92.4 and 98.8 Mbp). In addition to this, there is a QTL for stem strength (T18SBSL) [34] in this area, although it is located at a higher distance than the markers identified by GWAS in bread wheat.

Similarly, the marker DS.DW.1064412 (at 443.1 Mbp), close to IWA2972 (at 442.5 Mbp), is related to lodging tolerance. All the remaining MTAs for stem traits in chromosome 2B are located close (between 0.6 and 2 Mbp) to markers related to lodging tolerance.

The co-localization of the MTAs for culm wall thickness and stem diameter with lodging is in agreement with the positive correlation between these traits and reduced lodging (reviewed by [23]). The high degree of collinearity between Triticae species [36,37] explains that the same regions are controlling similar traits in both durum and bread wheat. Thus, the diversity for stem traits in durum wheat is readily available for bread wheat breeding through the development of new synthetic wheats.

In addition to the findings described above, stem properties are also associated with resistance to WSS. Varella et al. [20] reported several QTLs for WSS resistance, including a new for stem solidness on 6B, a QTL associated with stem solidness in early development on chromosome 3A (Qss.msub.3AL) and a QTL for WSS mortality on 3B. Stem properties are related to wheat stem sawfly resistance [16]. Thus, we investigated whether the MTAs identified in this work may co-localize with any of these QTLs. To do this, we considered the markers within the confidence intervals for the QTLs cited above as reported by Varella et al. [20]. We searched for these markers in the ‘Svevo’ genome (Supplementary Table S3). The QTL for WSS-mortality on 3B spans 97 cM in genetic distance, corresponding to 547 Mb in the ‘Svevo’ reference genome (Figure 6). The marker 5566511 was associated with pith area differences, and thus, it may indicate that the differences in WSS mortality reported by Varella et al. [20] could be related to differences in the pith area. On the contrary, no MTA was detected within the confidence interval of Qss.msub-3AL (Figure 6), or with the stem solidness gene located in chromosome 3B (QTL 1005; Figure 6) [15].

Regarding the QTL for late stem solidness on chromosome 6B [20], the search in the ‘Svevo’ genome returned unexpected results. Twenty markers from the confidence interval of this QTL were found, but only three of them were located in chromosome 6B (Supplementary Table S3). However, chromosome 2B of ‘Svevo’ contains many of these markers. This is relevant since the majority of the MTAs for stem traits identified in this work are located in this chromosome. The confidence interval for the stem solidness QTL spans 73 cM, which corresponds to 572 Mb. Five markers were simultaneously associated with pith area and stem area in two different regions within this chromosome (Figure 6). Four of them co-localized at the same position. Thus, the variation in these traits seems to be related to the variation reported by Varella et al. [20] for late stem solidness.

Previous studies have shown that landraces from the Iberian Peninsula (Spain and Portugal) show the greatest haplotype diversity in relation to WSS resistance [15]. Thus, landraces from this area are a good source of resistance against this pest. Indeed, all WSS-resistant hexaploid wheat varieties in North America, except ‘Conan’, carried the allele inherited from the Portuguese landrace ‘S-615′ [15]. The co-localization of stem cross section parameters with QTLs for WSS resistance is in agreement with the importance of stem traits for this resistance [16].

Reduced height has been important to reduce the likelihood of lodging. However, the opportunities for improving lodging tolerance by reducing plant height are limited since the minimum height for optimum grain yields is already being approached [38]. Accordingly, other plant traits, such as stem diameter, culm wall thickness or stem diameter, should be considered since they are associated with reduced lodging [23]. The MTAs for stem traits identified in this work that co-locate with previous QTLs can serve as a reference for the development of MAS strategies in wheat breeding. The diversity found in the plant material studied in this work highlights the potential of the plant genetic resources, as reported for other traits [11,12,14,39].

## 3. Materials and Methods

### 3.1. Plant Material, Field Testing and Statistical Analyses

The plant material conserved at the Centre for Plant Genetic Resources (CRF-INIA, Alcalá de Henares, Spain) was utilized for this work. A total of 150 accessions were evaluated, including the Spanish durum wheat core collection [39] (Supplementary Table S2). Additional passport data can be obtained from the Spanish Inventory of Plant Genetic Resources Centre [40].

### 3.2. Field Design and Statistical Analysis

The wheat collection was evaluated in an augmented block design during one season [41]. The design consisted of 13 blocks containing 12 genotypes in each, with 150 test entries and two check entries at Córdoba (Spain). Field experiments consisted of non-replicated rows of 1 m long with 10 plants per row. Plants were grown in field conditions that included anti-weed nets and an anti-bird net structure. In each block, the checks were allotted randomly.

All accessions were evaluated for the heading date (growing degrees day, gdd) (HD) and plant height. At maturity, all the stems were cut 5 cm above the surface, and a random sample of 10 stems per accession was selected. Stem section images from basal internodes were taken with a Canon PowerShot SX20 IS camera and were further analyzed with Software NIS_Elements, v. 4.50, Nikon Instruments Inc. for the assessment of the following traits: total stem area (SA) (mm^2^), pith area (PA) (mm^2^), pith diameter (PD) (mm) and culm wall thickness (CWT) (mm) (stem diameter—pith diameter)/2.

The analysis of variance was performed using the R package “AugmentedRCBD” [42], which is a function for analysis of variance of an augmented randomized block design [41,43] and the generation, as well as comparison, of the adjusted means of the treatments/genotypes. Computations for estimating heritability in single environments were based on the BLUEs of genotypic effects using Formula 19 from [44],

$$H^{2}=\frac{\sigma_{g}^{2}}{\sigma_{g}^{2}+v/2}$$

where *v* is the mean variance of the difference of two adjusted treatment means (BLUE). Correlograms were obtained using the BLUEs and GGally packages in RStudio.

### 3.3. DNA Isolation, Genotyping and Marker-Trait Associations

Genomic DNA was isolated from two-week-old leaves of seedlings following the CTAB protocol with slight modifications [45], using the TissueLyser II mill (Qiagen), two stainless-steel balls (5 mm diameter) for sample disruption and 2 mL Eppendorf tubes. Genotyping by sequencing analysis was performed at Diversity Arrays Technology Pty Ltd. (Canberra, Australia).

The DArtSeq markers were aligned to the durum wheat reference genome ‘Svevo’ [24]. A BLASTn search [46] was performed using BLAST+ [47] with the following criteria: E-value of <1.5 × 10^−6^ and sequence identity of > 80%. DArTSeq sequences were used as a query against the durum wheat coding sequences (nucleotides) of annotated high (HC) and low (LC) confidence genes. Only DArTSeq markers with a significant match to HC or LC genes were considered for association tests.

A principal component analysis (PCoA) was conducted based on genotype data from the DArTSeq markers using Tassel 5.2.45 [48] to inspect the existence of structures in the durum wheat collection. A PCoA biplot was depicted using the ggplot2 [49] and ggrepel packages in RStudio. Marker-trait associations were determined using TASSEL 5.2.45 [48]. Markers with a minimum allele frequency of less than 5% and 10% of missing data points were not included in the association analyses. Association analyses were performed using a mixed linear model (MLM), including the PCoA as the Q matrix and the kinship matrix was calculated with Tassel MLM (Q + K). The false discovery rate (FDR) for each trait was calculated with the approach developed by Benjamini and Hochberg [28] using the RainbowR package [50] and RStudio v. 1.2.1335 [51]. Manhattan plots were obtained using the qqman package [52] in RStudio.

### 3.4. Linkage Disequilibrium (LD) Decay

Pairwise marker correlations (r^2^ values) were calculated on the DArTSeq dataset using TASSEL 5.2.45 software [48]. The significance of the pairwise LD (*p* values) was computed using 1000 permutations. Inter-chromosomal pairs (unlinked loci) were used to determine a critical value of r^2^ using the 95th percentile of the distribution as the threshold, beyond which the LD is probably caused by a real linkage. Intra-chromosomal r^2^ values were plotted against the physical distance (Mbp), and a smooth line was fitted using the LOESS regression and R. The intersection between the LOESS curve and the critical threshold was used as the estimate of the extent of LD decay.

## 4. Conclusions

The co-localization of the MTAs identified in this work with those of previous studies (either QTLs or GWAS) in both durum and bread wheat confirms the importance of these markers in relation to stem traits. On the contrary, some MTAs would require further validation before they can be considered for MAS strategies. The involvement of syntenic regions of chromosome 2B in durum and bread wheat for the genetic control of stem traits and lodging suggests the existence of a common genetic basis in both species.

The diversity found in the durum wheat landraces indicates their potential as donors for stem-related traits. In this context, it is important to note the relevance of the activities developed at plant genetic resource centers, such as the CRF, for the conservation of plant genetic resources. Finally, the MTAs for stem-related traits identified in this work can serve as a reference for further development of markers allowing for the efficient introgression of target traits into elite material.

## Figures and Tables

**Table 1:** Marker-trait associations (MTAs) found for the selected phenotypic traits and DArTseq markers.

Marker ^1^	Trait	Chrm	Pos ^2^	LOD	FDR ^3^	R-Square	Effect ^4^
DS.DW.2290879	Stem area	2B	90.2	4.47	4.29	0.070	14.6
DS.DW.1064412	Stem area	2B	443.1	5.08	4.29	0.066	14.5
DS.DW.1716898	Stem area	2B	631.8	4.33	4.29	0.064	17.4
DS.DW.2299734	Stem area	2B	631.8	5.47	4.29	0.074	19.6
DS.DW.1315604C	Stem area	2B	631.8	4.59	4.29	0.075	18.4
DS.DW.995800	Stem area	2B	631.8	7.86	4.29	0.115	19.8
DS.DW.999492	Stem area	7A	20.0	4.64	4.29	0.079	16.5
DS.DW.12773467	Stem area	7A	714.7	4.59	4.29	0.055	14.2
DS.DW.2351188	Culm wall thickness	1A	34.8	4.65	4.63	0.091	1.6
DS.DW.2374725	Culm wall thickness	1B	52.5	4.75	4.63	0.089	1.7
DS.DW.995800	Culm wall thickness	2B	631.8	5.37	4.63	0.091	2.3
DS.DW.1094684	Culm wall thickness	3A	691.7	4.84	4.63	0.071	1.8
DS.DW.994979	Height	4A	606.0	4.8	4.79	0.063	40.7
DS.DW.5564719	Height	5A	500.0	5.17	4.79	0.060	41.3
DS.DW.1064412	Pith area	2B	443.1	5.5	4.2	0.129	10.1
DS.DW.1716898	Pith area	2B	631.8	4.34	4.2	0.084	11.4
DS.DW.2299734	Pith area	2B	631.8	4.31	4.2	0.088	10.9
DS.DW.1315604	Pith area	2B	631.8	5.36	4.2	0.128	12.4
DS.DW.995800	Pith area	2B	631.8	4.35	4.2	0.082	10.5
DS.DW.2284385	Pith area	2B	676.7	5.51	4.2	0.125	9.5
DS.DW.5566511	Pith area	3B	659.3	4.56	4.2	0.100	9.7
DS.DW.3064906	Pith area	5B	584.1	5.61	4.2	0.107	8.8
DS.DW.2307793	Pith area	6B	55.8	4.49	4.2	0.095	6.3
DS.DW.999492	Pith area	7A	20.0	5.99	4.2	0.138	11.9
DS.DW.2351188	Pith diameter	1A	34.8	4.61	4.61	0.107	2.0
DS.DW.2290879	Pith diameter	2B	90.2	4.83	4.61	0.085	2.5
DS.DW.1094684	Pith diameter	3A	691.7	5.85	4.61	0.091	2.4
DS.DW.1240805	Pith diameter	4A	633.7	6.05	4.61	0.088	3.1
DS.DW.3570185	Heading date	2B	788.1	4.34	ns	0.045	136.1

^1^ DS.DW. = DArTSeq Durum Wheat; ^2^ Position in Mbp; ^3^ False discovery rate at α = 0.05; ^4^ Difference in the effect between alternative alleles.

## Data Availability

Data are contained within the article.

## Supplementary Materials

The following are available online at https://www.mdpi.com/article/10.3390/plants10061123/s1, Table S1: DArTSeq markers with a significant match to high or low confidence gene models in the ‘Svevo’ genome, Table S2: List of accessions analyzed in this work, Table S3: Location of markers within the confidence intervals of selected QTLs reported by Varella et al. [20] in the ‘Svevo’ genome, Table S4: Location of markers associated with lodging and stem diameter shown in Figure 6.
